# Modulation of hepatic perfusion did not improve recovery from hepatic outflow obstruction

**DOI:** 10.1186/s40360-017-0155-4

**Published:** 2017-06-26

**Authors:** J. Arlt, W. Wei, C. Xie, A. Homeyer, U. Settmacher, U. Dahmen, O. Dirsch

**Affiliations:** 1 Experimental Transplantation Surgery, Department of General, Visceral and Vascular Surgery, Jena University Hospital, Drackendorfer Str. 1, 07747 Jena, Germany; 2 Fraunhofer Institute for Medical Image Computing MEVIS, Universitätsallee 29, 28359 Bremen, Germany; 3 Department of General, Visceral and Vascular Surgery, Jena University Hospital, Erlanger Allee 101, 07747 Jena, Germany; 4 Institute of Pathology, Klinikum Chemnitz, Flemmingstraße 2, 09116 Chemnitz, Germany

**Keywords:** Liver resection, Portal hypertension, Vasoactive drugs, Splenectomy

## Abstract

**Background:**

Focal hepatic venous outflow obstruction frequently occurs after extended liver resection and leads to a portal hypertension, arterial hypoperfusion and parenchymal necrosis.

In this study, we investigated the pharmacological modulation of liver perfusion and hepatic damage in a surgical model of hepatic outflow obstruction after extended liver resection by administration of 5 different drugs in comparison to an operative intervention, splenectomy.

**Methods:**

Male inbred Lewis rats (Lew/Crl) were subjected to right median hepatic vein ligation + 70% partial hepatectomy. Treatment consisted of a splenectomy or the application of saline, carvedilol or isosorbide-5-mononitrate (ISMN) (5 mg · kg^−1^ respectively 7,2 mg · kg^−1^ per gavage 12 h^−1^). The splenectomy was performed during operation. The effect of the treatments on hepatic hemodynamics were measured in non-operated animals, immediately after operation (*n* = 4/group) and 24 h after operation (*n* = 5/group). Assessment of hepatic damage (liver enzymes, histology) and liver cell proliferation (BrdU-immunohistochemistry) was performed 24 h after operation. Furthermore sildenafil (10 μg · kg^−1^ i.p. 12h^−1^), terlipressin (0.05 mg · kg^−1^ i.v. 12 h^−1^) and octreotide (10 μg · kg^−1^ s.c. 12 h^−1^) were investigated regarding their effect on hepatic hemodynamics and hepatic damage 24 h after operation (*n* = 4/group).

**Results:**

Carvedilol and ISMN significantly decreased the portal pressure in normal non-operated rats from 11,1 ± 1,1 mmHg (normal rats) to 8,4 ± 0,3 mmHg (carvedilol) respectively 7,4 ± 1,8 mmHg (ISMN). ISMN substantially reduced surgery-induced portal hypertension from 15,4 ± 4,4 mmHg to 9,6 ± 2,3 mmHg. Only splenectomy reduced the portal flow immediately after operation by approximately 25%. No treatment had an immediate effect on the hepatic arterial perfusion. In all treatment groups, portal flow increased by approximately 3-fold within 24 h after operation, whereas hepatic arterial flow decreased substantially. Neither treatment reduced hepatic damage as assessed 24 h after operation. The distribution of proliferating cells appeared very similar in all drug treated groups and the splenectomy group.

**Conclusion:**

Transient relative reduction of portal pressure did not result in a reduction of hepatic damage. This might be explained by the development of portal hyperperfusion which was accompanied by arterial hypoperfusion.

**Electronic supplementary material:**

The online version of this article (doi:10.1186/s40360-017-0155-4) contains supplementary material, which is available to authorized users.

## Background

Extended hepatic resections are performed to remove liver tumors and in living liver donation. Extended hepatic resection leads to a massive reduction of liver mass by 60 to 70%.

The function of the remnant liver is determined by the optimal balance of blood inflow and outflow. The balance of blood inflow from both portal vein and hepatic artery is regulated through the hepatic arterial buffer response. The reduction of liver mass leads to an increase of portal venous flow, subsequently to portal hypertension and further to portal hyperperfusion [1, 2]. The portal hyperperfusion induces an arterial hypoperfusion and inhomogeneous perfusion in the liver [3]. An extended liver resection with transection of the middle hepatic vein results in a focal hepatic venous outflow obstruction (FHOO). The obstruction leads to the formation of necrosis in the undrained tissue. This results in an additional loss of functional liver mass in the remnant liver [4].

In our previous study, we developed a surgical model of extended hepatic resection in Lewis rats (Lew/Crl) [4]. In this surgical model, we induced FHOO by a right median hepatic vein ligation and combined this procedure with 70% partial hepatectomy. We observed that portal hypertension resulting from reducing the size of the liver impaired the spontaneous recovery from FHOO. In a subsequent study, we observed that hepatic arterial perfusion determined the extent of hepatic necrosis, the formation of vascularized sinusoidal canals and the kinetic of parenchymal recovery [5]. We attempted to modulate hepatic perfusion by influencing nitric oxide metabolism. Nitric oxide (NO) is known to modulate the hepatic arterial perfusion and mediate hepatic regeneration. Inhibition of NO-release by administrating L-N^G^-Nitroarginine methyl ester (L-NAME), an analog of arginine, aggravated liver damage. In contrast, the NO-release induced by the application of molsidomine, a NO donor, did not have a beneficial effect in terms of liver damage and hepatic regeneration despite a measurable effect on hepatic hemodynamics [6].

In clinical practice splenectomy is performed as operative intervention to reduce portal hyperperfusion [7]. However, this additional surgical procedure increases the over-all risk for complications [8–10].

The objective for this study was to investigate the modulation of hepatic hemodynamics after an extended hepatic resection by applying five clinically used drugs: We investigated the modulation of portal hemodynamics after an extended hepatic resection by applying the beta-blocker carvedilol, the nitrovasodilator isosorbide-5-mononitrate (ISMN), the vasopressin analogue terlipressin and the long-acting somatostatin analogue octreotide [11–14]. We investigated the modulation of arterial hypoperfusion after an extended hepatic resection by applying the phosphodiesterase-5 inhibitor sildenafil [15].

## Methods

### Experimental design

This study was divided in two parts. In both parts, Lewis (Lew/Crl) rats were subjected to right median hepatic vein ligation + 70% partial hepatectomy. In the first part, treatment consisted of a splenectomy or the application of saline (PZN-1636349; Fresenius Kabi, Bad Homburg, Germany), carvedilol (PHR1265 Fluka, Sigma-Aldrich Co., St. Louis, MO, USA) or ISMN (I0775010 Fluka, Sigma–Aldrich Co., St. Louis, MO, USA) (5 mg · kg^−1^ respectively 7.2 mg · kg^−1^ per gavage). The splenectomy was performed during the extended liver resection. In the first group, the effect of the treatments on hepatic hemodynamics was measured in non-operated animals and immediately after extended liver resection (group 0 h; *n* = 4). In the second group, the effect of the treatments on hepatic hemodynamics was measured 24 h after the operation (group 24 h, *n* = 5). The assessment of hepatic damage was performed 24 h after the operation.

In the second part of this study, based on these results, we investigated the effect of the remaining drugs sildenafil (CAYM14008-50, CAYMAN CHEMICAL, Hamburg, Germany) (10 μg · kg^−1^ i.p.), terlipressin (Hämopressin ®, Acorus Therapeutics LTD, Chester, United Kingdom) (0.05 mg · kg^−1^ i.v.) and octreotide (Sandostatin®, Novartis, Basel, Switzerland) (10 μg · kg^−1^ s.c.) only 24 h after the operation (*n* = 4/group). All drugs were administered every 12 h in the 24 h experiments.

### Animals

Male inbred Lewis (Lew/Crl) rats (230–410 g, Charles River, Sulzfeld, Germany) were used in this study. Rats were fed a laboratory diet with water and rat chow ad libitum until harvest and were kept under constant environmental conditions with a 12 h light–dark cycle in a conventional animal facility using environmentally enriched type IV cages in groups of 2–3 rats. All procedures, experiments and housing of the animals were carried out according to current German regulations and guidelines for animal welfare and to international principles of laboratory animal care, following the ARRIVE Guidelines Checklist as well. The protocols were approved by the Thüringer Landesamt für Verbraucherschutz, Thuringia, Germany (Approval-Number: 02-023/14).

### Surgical procedures

All surgical interventions were performed under inhalation of 3% isoflurane mixed with pure oxygen at a flow of 0.5 L/min (isoflurane vaporizer, Sigma Delta, UNO, Zevenaar, Holland) in a dedicated S1 operation room. All instruments were thoroughly cleaned and tip-sterilized (tip-sterilizer, Cellpoint Scientific Inc., USA) before operation. After all operations, the abdominal incision of the rats was closed in two layers with running sutures using 3–0 prolene suture (Ethicon, Somerville, USA). The animals received buprenorphine for analgesic treatment in a dose of 0,05 mg · kg^−1^ body weight (Temgesic, RB Pharmaceuticals Ltd, UK).

#### Extended liver resection

The right median hepatic vein was exposed, isolated from liver mass and ligated with one 6–0 silk suture (Resorba, Nuremberg, Germany). Partial hepatectomy was performed employing a modified resection technique described in detail previously [6]. In brief, the left lateral lobe, the left median lobe, the superior plus inferior caudate lobes, and the right superior plus inferior lobes were resected, leading to an estimated 70% reduction of liver mass.

#### Splenectomy

The splenectomy was initiated by dissecting the gastrosplenic ligament, the phrenicocolic ligament and the splenorenal ligament. The splenic artery and vein were clamped and the spleen removed. Then the vessels were ligated close to the clamp using 4–0 silk sutures.

#### Hemodynamic measurement

In the groups of 0 h observation time, the hemodynamic parameters (mean arterial pressure (MAP), portal vein pressure (PVP) and portal flow (PVF), hepatic arterial flow (HAF)) were monitored before and after extended liver resection and in addition after splenectomy in the splenectomy group. In the groups of 24 h observation time, the hemodynamic parameters were monitored during the harvest procedure after 24 h observation time.

The hemodynamic parameters were recorded by Powerlab system (Powerlab 16/30; ADI Instruments Castle Hill, NSW, Australia) which was described in detail previously [16]. In brief, MAP was measured with a Millar Mikro-Tip® catheter transducer (Millar Instruments INC, Houston, USA) in the left carotid artery. MAP was recorded during the whole operation procedure. PVP was recorded during the whole operation procedure in the 0 h group using a 26G fluid-filled catheter inserted into the mesenteric vein. The PVF and HAF were determined using the MA2PSB flow probe respectively the MA0.5PSB flow probe. HAF was recorded till a stable value was obtained. The software Labchart 7 (ADInstruments Pty Ltd, Australia) was used for recording the data.

#### Harvesting and sampling procedure

One hour before the organ-harvesting procedure, 5-bromo-2-deoxyuridine (BrdU) (Sigma-Aldrich, St. Louis, MO) (50 mg/kg) dissolved in saline solution (0.9% NaCl) was injected intravenously. Serum was obtained via puncture of inferior vena cava using serum tubes (serum Z/1.2 ml, monovette, SARSTEDT) and was stored at −20 °C until measuring the liver enzymes. Liver tissue was collected for histological analysis in 4.5% buffered formalin.

### Liver damage

#### Liver enzymes

The serum level of aspartate transaminase (ASAT), alanine transaminase (ALAT) and total bilirubin were measured using Automated Chemical Analyser (Bayer Advia 1650, Leverkusen, Germany).

#### Liver histology

The formalin fixed liver tissue was embedded in paraffin and cut in 4 μm thick sections. Thereafter, slides were stained with haematoxylin-eosin (HE) or immunohistochemically for the detection of BrdU-incorporating cells [17]. All stained sections were scanned using a NanoZoomer 2.0 HT scanner and the software NDP.scan 2.3 from Hamamatsu Photonics K.K. (Hamamatsu City, Japan).

The extension of necrosis and viable hepatocytes (“viable portal islands”) in the obstruction zone (OZ) was evaluated in the scanned sections using the Histokat software (Fraunhofer MEVIS, Bremen, Germany, available on request*). The software uses machine-learning methods in order to identify necrotic tissue by colour and texture features. A detailed description of the utilized algorithm and its application is given in Arlt et al. and Homeyer et al. [18, 19].

The assessment of cell proliferation was performed by reading the digitalized slides using the software NDP.view2 from Hamamatsu Photonics K.K. (Hamamastu City, Japan). We assessed the pattern of BrdU-positive cells in respect to the zonation of the liver lobule and the localization of the confluent necrosis.

### Liver regeneration

The remnant liver was weighed for calculating the liver weight-to-body weight ratio (LW/BW) and liver lobe weight recovery. The LW/BW was calculated following the formula:

$$ \mathrm{L}\mathrm{W}/\mathrm{B}\mathrm{W}\left[\%\right] = \mathrm{remnant}\ \mathrm{liver}\ \mathrm{weight}\ \left[\mathrm{g}\right]/\mathrm{original}\ \mathrm{body}\ \mathrm{weight}\ \left[\mathrm{g}\right]\cdot 100\% $$

The liver weight recovery was calculated following the formula:

$$ \mathrm{Liver}\ \mathrm{weight}\ \mathrm{recovery}\ \left[\%\right] = \mathrm{remnant}\ \mathrm{liver}\ \mathrm{weight}\ \left[\mathrm{g}\right]/\mathrm{calculated}\ \mathrm{original}\ \mathrm{liver}\ \mathrm{weight}\ \left[\mathrm{g}\right]\cdot 100\% $$

### Statistical analysis

Results were expressed as mean ± standard deviation (SD) based on the demonstration of the normal distribution of the numerical data using the Shapiro-Wilk test by SPSS Statistics version 24.0 (IBM Corp., Armonk, NY, USA). Statistical analysis between multiple groups was performed using the Student’s *t*-test by SPSS Statistics version 24.0. Results were considered statistically significant with p-values of less than 0.05 were obtained (* *P* < 0.05).

## Results

All animals tolerated the procedure well, body weight loss ranged from 2 to 9,8% without significant differences between the groups at 24 h post-OP.

### Hemodynamic measurement

MAP remained stable throughout the operation in all animals and was not affected by the liver resection itself (variability around 3 ± 1 mmHg).

The hemodynamic measurements showed that average PVP of normal rats was 12,2 ± 2,4 mmHg (9,93 mmHg to 15,54 mmHg). Carvedilol and ISMN significantly reduced the PVP in normal non-operated rats resulting in a mean of 8,4 ± 0,3 mmHg (*P* < 0,03) respectively 7,4 ± 1,8 mmHg (*P* < 0,03) (Table 1).

**Table 1:** Changes in the hemodynamic parameters before, immediately and 24 h after operation

	0 h pre-OP			0 h post-OP			24 h post-OP		
	PVP [mmHg]	PVF [ml/min · g]	HAF [ml/min · g]	PVP [mmHg]	PVF [ml/min · g]	HAF [ml/min · g]	PVP [mmHg]	PVF [ml/min · g]	HAF [ml/min · g]
Saline	12,2 ± 2,4	1,3 ± 0,2	0,2 ± 0,0	15,4 ± 4,4	1,2 ± 0,4	0,2 ± 0,1	12,3 ± 2,7	3,3 ± 0,5	0,01 ± 0,0
Splenectomy	12,2 ± 2,4	1,3 ± 0,2	0,2 ± 0,0	14,3 ± 4,4	0,9 ± 0,2*	0,1 ± 0,1	11,0 ± 1,3	2,9 ± 1,0	0,02 ± 0,0
Carvedilol	8,4 ± 0,3*	1,4 ± 0,2	0,2 ± 0,1	12,0 ± 0,6	1,3 ± 0,1	0,2 ± 0,0	11,2 ± 1,8	4,3 ± 1,1	0,02 ± 0,0
Isosorbide-5-mononitrate	7,4 ± 1,8*	1,4 ± 0,1	0,3 ± 0,1	9,6 ± 2,3	1,2 ± 0,4	0,2 ± 0,1	10,6 ± 2,9	3,7 ± 1,7	0,06 ± 0,1
Sildenafil	-	-	-	-	-	-	11,8 ± 2,3	4,1 ± 1,9	0,01 ± 0,0
Terlipressin	-	-	-	-	-	-	12,8 ± 1,2	4,0 ± 1,5	0,03 ± 0,0
Octreotide	-	-	-	-	-	-	9,9 ± 2,3	3,7 ± 0,7	0,03 ± 0,0

* *P* < 0.05 compared to saline group

**Fig. 1:**
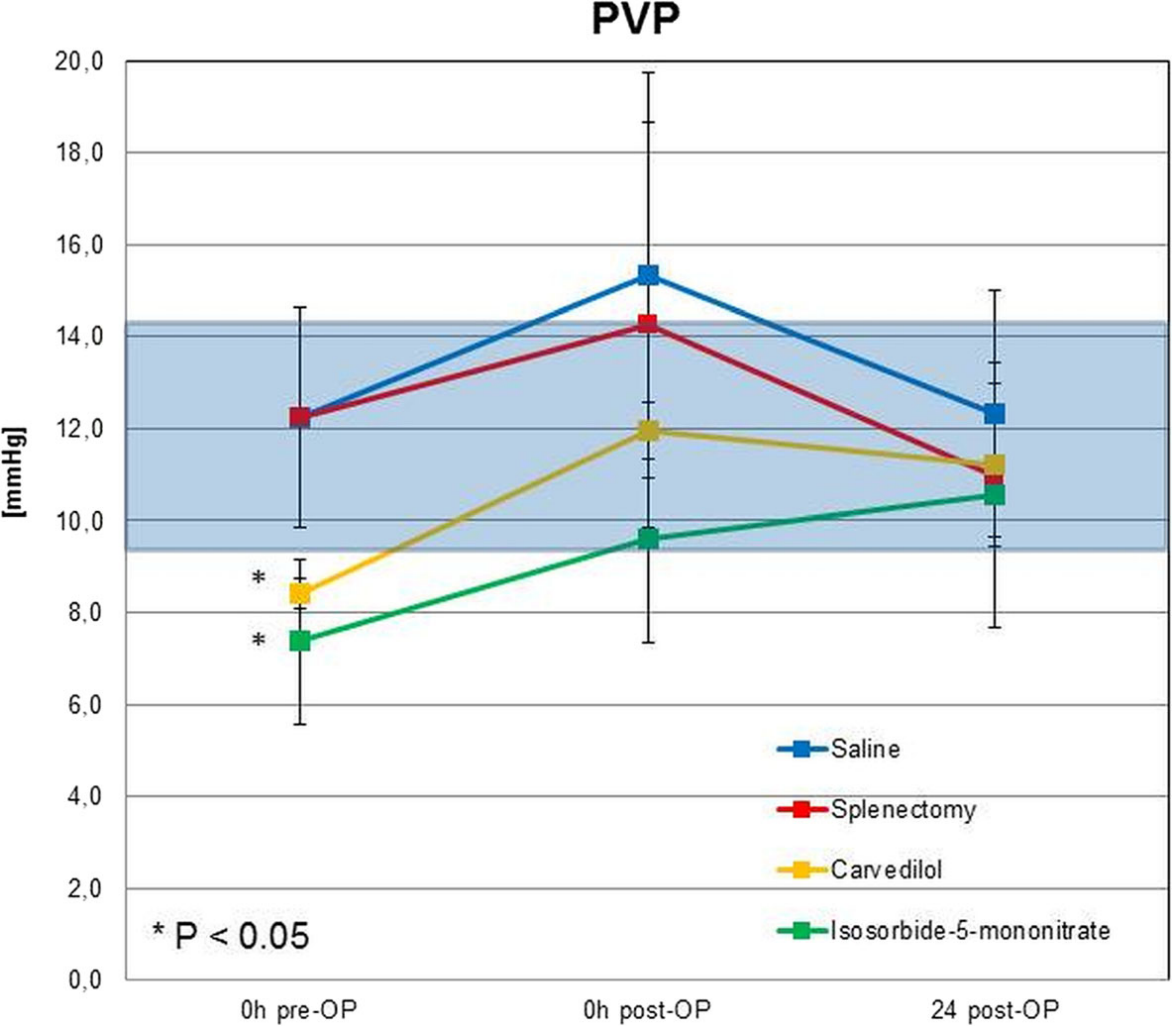
Portal vein pressure (PVP) in saline group, splenectomy group, carvedilol and isosorbide-5-mononitrate group (*n* = 4–5/group; *P < 0 .05 treatment group compared with saline group at 0 h pre-OP)

The hemodynamic measurements showed that the surgical intervention consisting of right median hepatic vein ligation + 70% partial hepatectomy induced portal hypertension leading to a PVP of 15,4 ± 4,4 mmHg. Both drugs, Carvedilol and ISMN, resulted in a substantial reduction in surgery-induced portal hypertension. ISMN application reduced mean PVP from 15,4 ± 4,4 mmHg to 9,6 ± 2,3 mmHg. Carvedilol administration and splenectomy reduced the PVP only slightly reaching a mean of 12,0 ± 0,6 mmHg (*P* = 0,200) respectively 14,3 ± 4,4 mmHg (*P* = 0,686) after the operation (Table 1; Fig. 1).

We observed a delayed effect of the surgical procedure on hepatic blood flow. Immediately after surgery, PVF and HAF remained in the same range as before. Only splenectomy reduced the PVF immediately after operation by approximately 25% from 1,2 ± 0,4 ml/min · g to 0,9 ± 0,2 ml/min · g (*P* < 0,03).

**Fig. 2:**
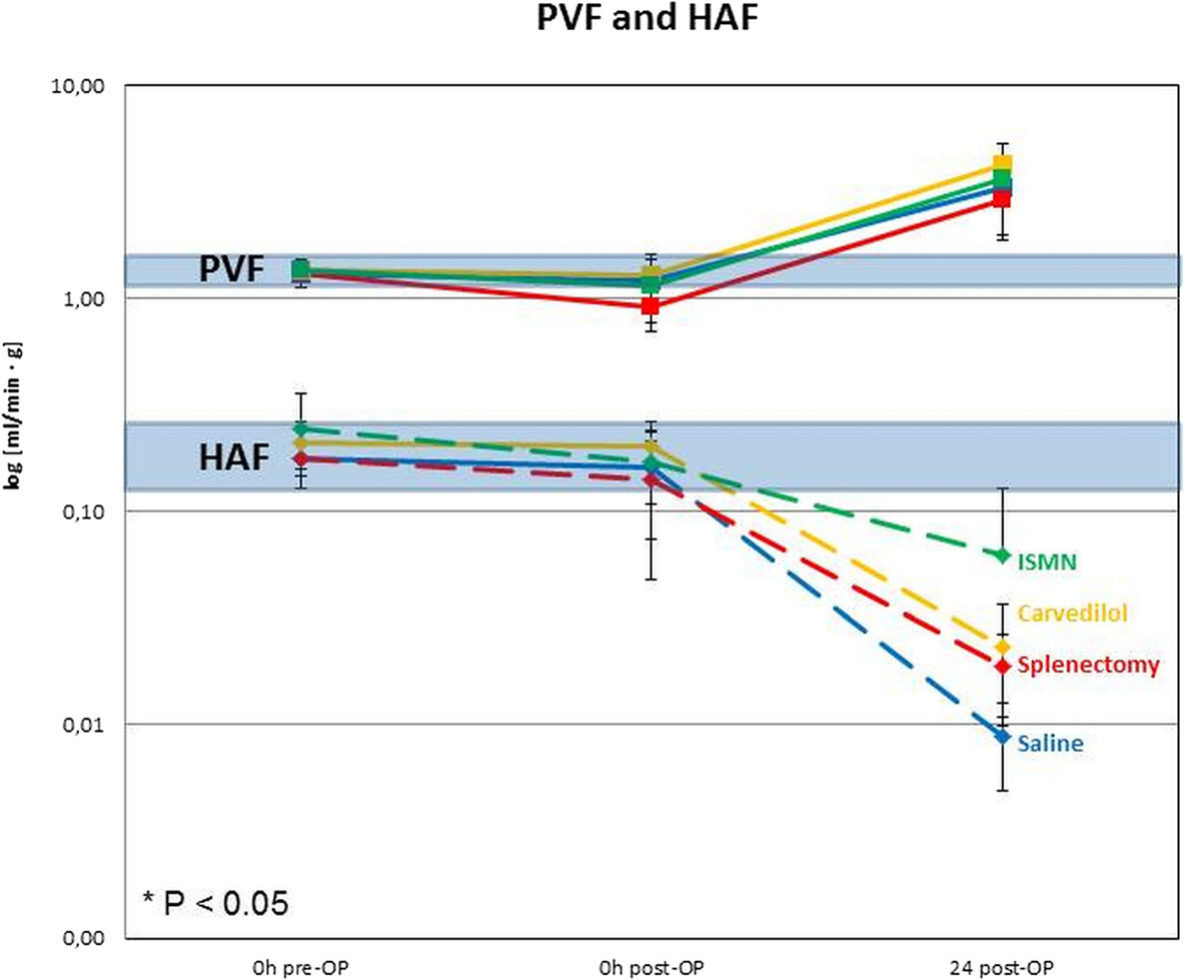
Portal vein flow (PVF) and hepatic arterial flow (HAF) in saline group, splenectomy group, carvedilol and isosorbide-5-mononitrate group (*n* = 4–5/group)

The hemodynamic measurements showed that 24 h post-OP the PVF increased by approximately 3-fold in all experimental groups, whereas HAF substantially decreased from 0,2 ± 0,1 ml/min · g to 0,01 ± 0,0 ml/min · g (Table 1; Fig. 2).

### Liver damage

**Fig. 3:**
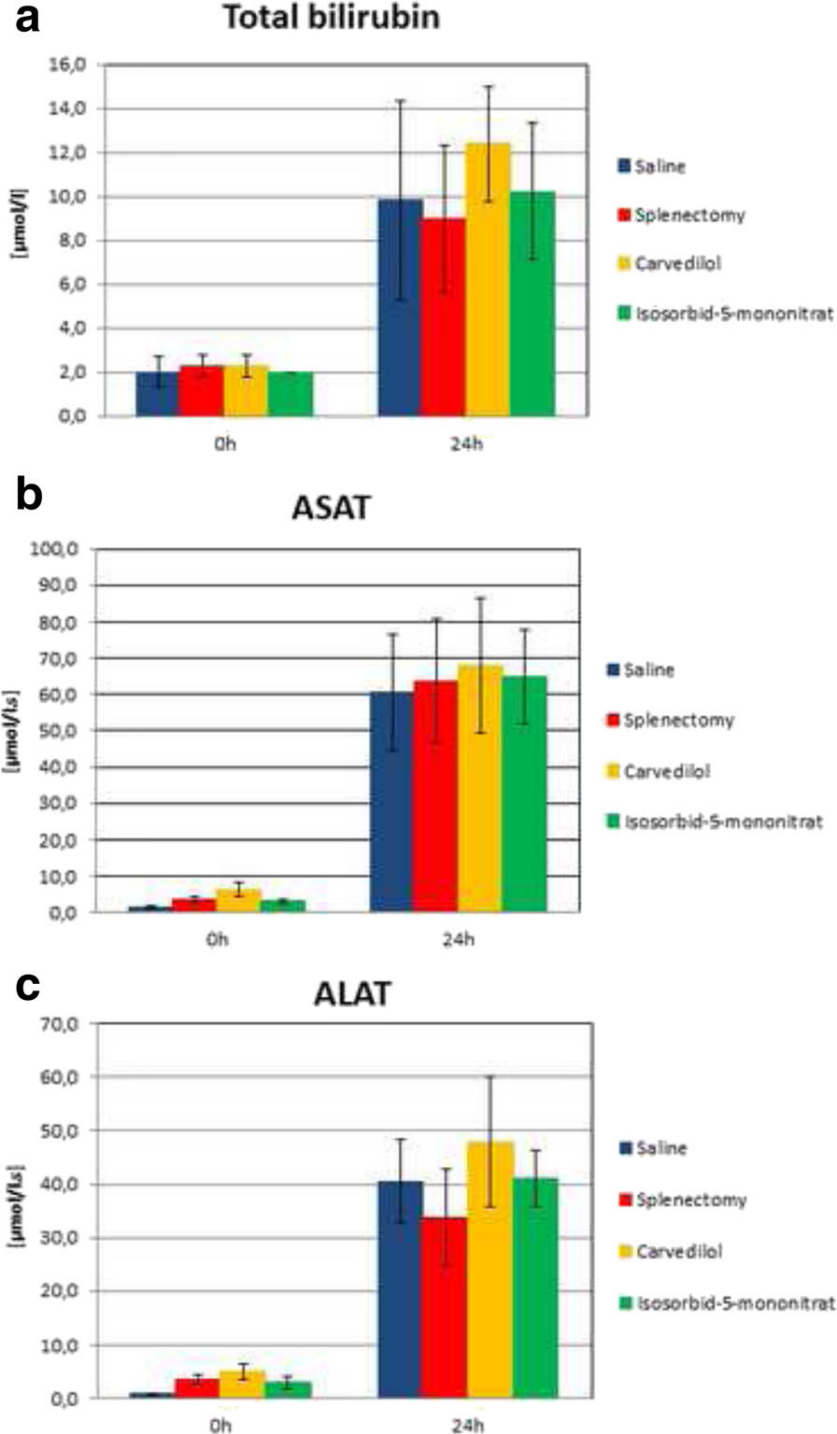
Serum liver enzyme level of **a** total bilirubin, **b** aspartate transaminase (ASAT) and **c** alanine transaminase (ALAT) and in saline group, splenectomy group, carvedilol and isosorbide-5-mononitrate group (*n* = 4–5/group)

Hepatic damage as indicated by the release of liver enzymes (aspartate transaminase (ASAT), alanine transaminase (ALAT)) and total bilirubin occurred within 24 h after the operation. Liver enzymes increased approximately 10-fold in all groups 24 h post-OP in comparison to 0 h post-OP respectively before surgery (Fig. 3).

Hepatic outflow obstruction seemed to represent a major injury driving cells in a state of unrecoverable injury finally resulting in ischemic cell death and confluent pericentral necrosis, irrespectively of the treatment.

**Fig. 4:**
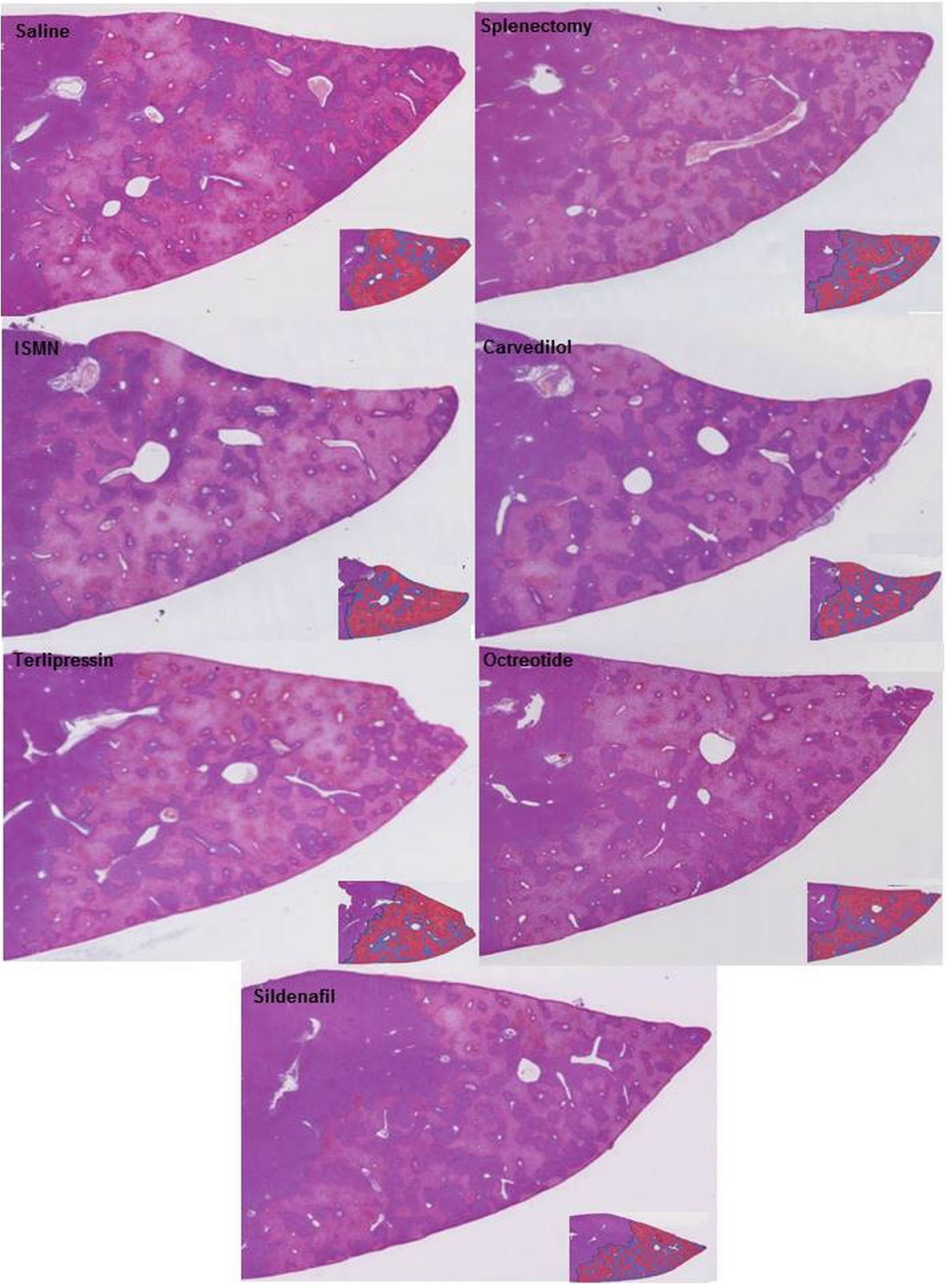
Images from HE-stained sections for histological evaluation of rim of viable hepatocytes in the obstructed zone (OZ) in saline group, splenectomy group, carvedilol, isosorbide-5-mononitrate (ISMN) group, terlipressin group, octreotide group and sildenafil group (×50). Small images on the right side show the HE-stained sections analysed with Histokat (rim of viable hepatocytes – blue; necrosis areas – red)

We observed in the obstruction zone large predominantly pericentral confluent necrosis covering zone 3 and zone 2 in all treatment groups at 24 h post-OP. The relative extension of confluent necrosis reached 58,6 ± 10,0% of the total surface of the OZ and covered zone 3 and zone 2. The relative size of the necrotic areas did not differ substantially, suggesting that the primary injury was rather severe (Fig. 4).

In all groups observed for 24 h post-OP, portal tracts were surrounded by a rim of viable hepatocytes (“viable portal islands”). These “viable portal islands” were surrounded by large confluent pericentral necrosis, as previously described [4].

### Liver regeneration

Liver weight-to-body weight ratio (LW/BW) reached approximately 1,7 ± 0,2% in all groups 24 h post-OP. The liver weight recovery was also similar in all groups 24 h after right median hepatic vein ligation + 70% partial hepatectomy and reached approximately 55 ± 2,5% of the calculated original liver weight.

**Fig. 5:**
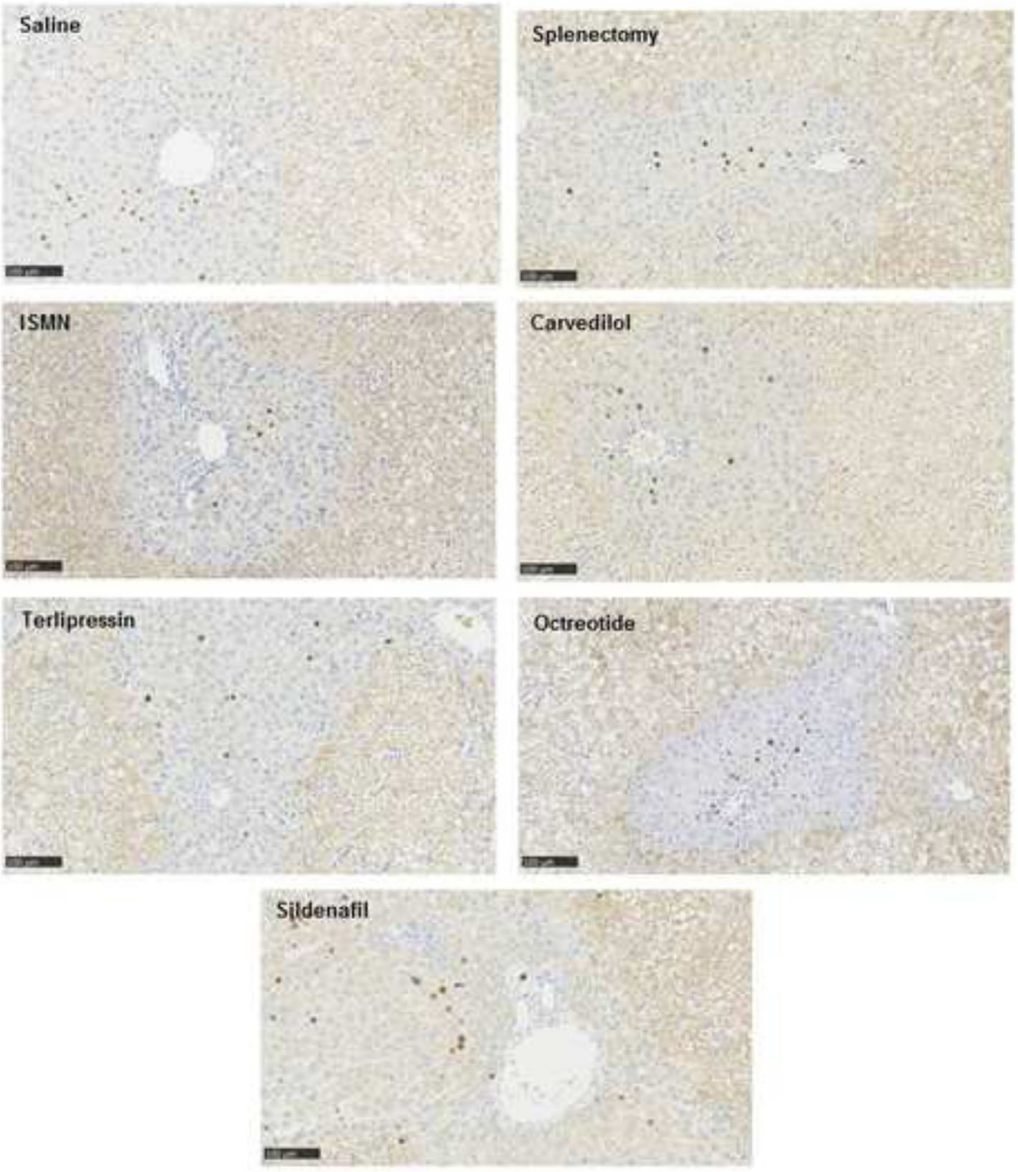
Images from BrdU-stained sections for histological evaluation of liver cell proliferation in the obstructed zone (OZ) in saline group, splenectomy group, carvedilol, isosorbide-5-mononitrate (ISMN) group, terlipressin group, octreotide group and sildenafil group (×1000)

In all groups observed for 24 h post-OP, we observed hepatocyte proliferation in OZ. The proliferating cells were located in zone 1 surrounding the portal field. The distribution of proliferating cells appeared very similar in all drug treated groups and the splenectomy group (Fig. 5).

## Discussion

The loss of functional liver mass after an extended liver resection is dependent on the balance between portal and arterial perfusion. The surgical reduction of liver mass leads to an increase of portal pressure (portal hypertension) and an increase of portal venous flow (portal hyperperfusion) and subsequently to arterial hypoperfusion. We hypothesized that the decrease of portal hypertension and the improvement of the balance between portal and arterial perfusion could limit the extent of damage after an extended liver resection [4, 5].

In this study, we aimed at evaluating the effect of surgical and pharmacologic modulation of hepatic hemodynamics on the extent of hepatic damage in a rat model of hepatic outflow obstruction.

**Fig. 6:**
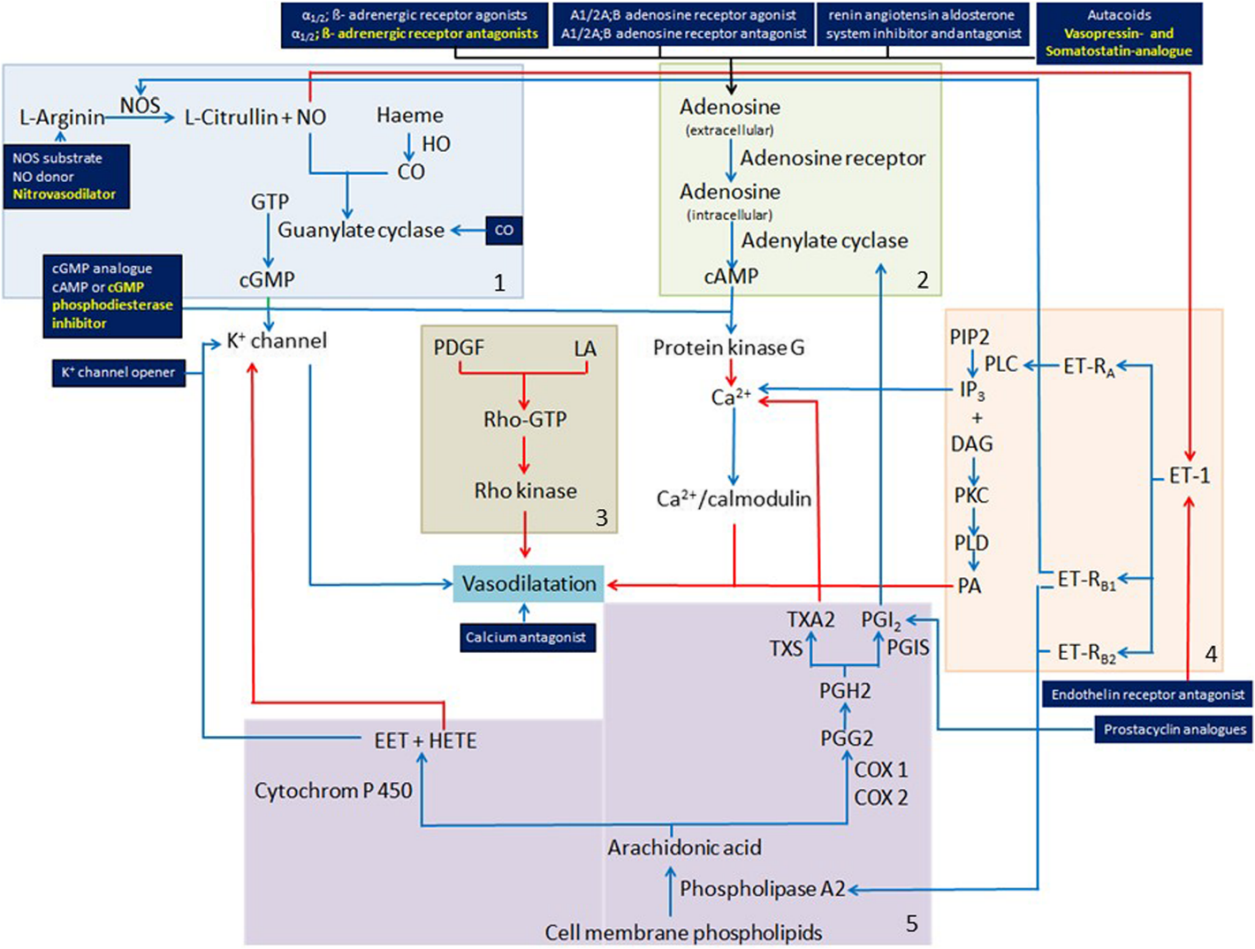
Diagram of different molecular vasoactive pathways involved in the regulation of hepatic hemodynamics with more than 20 drug classes. **List of interactions between pathways**: blue arrows - enhancing interactions; red arrows - inhibitory interactions; black arrows - interactions depending on drug class; dark blue tiles: interrelated drug classes; yellow text - drug classes with substance investigated in this study; white text - drug classes not investigated in this study. **List of abbreviations**: Ca^2+^- Calcium; cAMP - cyclic adenosine monophosphate; cGMP - cyclic guanosine monophosphate; COX 1/2 -Cyclooxygenase-1/2; DAG – Diacylglycerol; EET- Epoxyeicosatrienoic acids; ET-1-Endothelin 1; ET-R_A_-Endothelin receptor type A; ET-R_B1/2_ - Endothelin receptor type B 1/2; GTP - Guanosine triphosphate; HETE - Hydroxyeicosatetraenoic acids; HO - Haeme oxygenase; IP_3_ - Inositol 1,4,5-trisphosphate; K^+^ -Potassium; LA - Lysophosphatidic acid; NO - Nitric oxide; NOS - Nitric oxide synthetase; PA- Phosphatidic acid; PDGF - Platelet-derived growth factor; PGH2 - Prostaglandin H2; PGI_2_ - Prostacyclin; PGIS -Prostacyclin synthase; PIP2 - Phosphatidylinositol 4,5-bisphosphate; PKC- Protein kinase C; PLC -Phospholipase C; PLD - Phospholipase D; TXA2- thromboxane A2; TXS- Thromboxane synthase. **List of references contributing to the development of the diagram**: PMID:3904482; PMID:11889082; PMID:16518376; PMID:19789382; PMID:3356403; PMID:18484616; PMID:22468079; PMID:7938175; PMID:2194921; PMID:18346684; PMID:12846445; PMID:10728801; PMID:8253109

As surgical modulation of portal hemodynamics, we performed a splenectomy. For pharmacologic modulation of portal hemodynamics, we selected four drugs: carvedilol, ISMN, terlipressin or octreotide. For modulation of arterial hypoperfusion we used sildenafil. We selected the drugs because of their clinical effects and involvement in different vasoactive pathways (Fig. 6).

### Surgical modulation of portal hemodynamics

In clinical practice, reduction of splenic blood flow by splenectomy or ligation of the splenic artery is performed as surgical intervention to reduce portal hyperperfusion. However, splenectomy is an additional surgical procedure that might increase the over-all risk for complications [7–10]. As reported by Ikegami et al., complications after splenectomy consist of but are not limited to pancreatic leakage, splenic vein thrombosis and overwhelming post-splenectomy sepsis [7, 10]. They reported a complication rate of 10.1% (9/89) after living donor liver transplantation [10]. Winslow et al. observed an even higher complication rate of 26,6% including pulmonary, wound and infectious complications [8].

In experimental studies, controversial results were reported regarding the effect of splenectomy on portal venous flow. In our study, splenectomy reduced the PVF immediately after right median hepatic vein ligation + 70% partial hepatectomy by approximately 25%. Glanemann et al. also observed that the splenectomy decreased the recipient portal inflow by approximately 35% after 90% partial hepatectomy [20]. In contrast, splenectomy did not induce a measurable reduction in PVF after 70 to 90% partial hepatectomy as reported by Ogata et al., Darnis et al. and Eipel et al. [21–23].

Subsequently to the reduction of the portal flow to the liver the portal venous pressure should decrease. The decrease of portal venous pressure was documented in different clinical studies using splenectomy after living donor liver transplantation or in patients with liver cirrhosis [7, 24–26]. Also Yoichi et al. and Kuriyama et al. described that splenectomy significantly decreased the PVP after partial liver transplantation in rats (see Additional file 1: Table S1) [27, 28].

However, splenectomy did not lead to a substantial decrease of portal venous pressure in our experimental study. This result was confirmed by other experimental studies applying splenectomy after partial hepatectomy. As observed in our study splenectomy did not significantly reduce PVP after 84% PH in dogs, 70%PH and 90% PH in swine (see Additional file 1: Table S1) [21, 22].

Based on the theory of the hepatic arterial buffer response reduction of portal flow should lead to an increase of hepatic arterial flow. Eipel et al. reported that splenectomy did not affect HAF when performing a liver resection of up to 70% partial hepatectomy but did have an effect when reducing the liver mass by more than 85% partial hepatectomy [23]. Our operation consisting of right median hepatic vein ligation + 70% partial hepatectomy leads to a loss of approximately 80% of the liver mass due to the approximately 10% additional loss of functional liver mass because of the pericentral necrosis [4]. However, splenectomy did not affect HAF after the loss of approximately 80% of the liver mass in our model. This result confirms the observation of Eipel et al. who reported that the effect on HAF was related to the loss of liver mass [23]. Apparently, this regulatory process is only initiated after the loss of liver mass exceeds a cut-off which must be above 80% (see Additional file 1: Table S1).

### Pharmacological modulation of hepatic hemodynamics

The pharmacological modulation of hepatic hemodynamics by vasoactive drugs might prevent the additional surgical risk caused by an additional surgical intervention such as splenectomy [6, 29]. By means of knowledge-based selection, we chose five substances out of more than 20 drug classes addressing different molecular pathways (Fig. 6). All drugs were selected based on their described effect to either improve the balance between portal and arterial perfusion or/and to reduce portal hypertension as reported in clinical studies and experimental investigations. The effect of drugs on hepatic hemodynamics was mostly observed in acute experiments of less than 8 h observation time and rarely over an extended observation period as in our study.

We investigated the effect of the drugs on hepatic perfusion, portal pressure and hepatic damage after right median hepatic vein ligation + 70% partial hepatectomy immediately and 24 h after operation. The dose, route and timing of application of the drugs were selected based on previous publications and the reported half-life of drugs ranging from 5 to 8 h [30–34].

### Pharmacological modulation of portal hypertension

The pharmacological modulation of portal hypertension was investigated by the administration of ISMN and carvedilol.

### ISMN

The nitrovasodilator ISMN reduces systemic and splanchnic blood flow based on peripheral venous pooling and reduced cardiac preload. Additionally, ISMN releases NO which is active in the NO pathway. NO acts as hepatic vasodilator and modulates the liver sinusoidal tone. Both effects are supposed to reduce portal hypertension and activate HABR (Fig. 6 box 1) [35].

Reduction of portal hypertension was shown in patients with liver cirrhosis in different clinical studies, for example, Gargia-Pagán et al. described that ISMN decreased portal hypertension and increased HAF in patients with cirrhosis [12, 36–38]. Furthermore, the administration of ISMN led to a decrease of the hepatic venous pressure gradient within 40 to 60 min after application (see Additional file 1: Table S2) [36, 39].

Salam et al. reported that a dose of 7,2 mg · kg^−1^ ISMN applied by gastric gavage protected rats against hepatic injury and hepatocellular necrosis induced by carbon tetrachloride (see Additional file 1: Table S2) [31]. Therefore, we assumed that ISMN could also reduce the development of large confluent hepatocellular necrosis caused by outflow obstruction after extended liver resections and RHMV ligation [4].

In our study, the administration of ISMN decreased the PVP in normal rats. However, only ISMN reduced the surgery-induced portal hypertension immediately after right median hepatic vein ligation + 70% partial hepatectomy. The reduction of portal hypertension by ISMN did not have an effect on PVF and HAF. These observations are in contrast to the clinical observations described above.

### Carvedilol

Carvedilol is a potent non-cardioselective beta-blocker. Carvedilol blocks the alpha-1 receptors on the smooth muscles of the arterial vasculature. Based on the blockade of alpha-1 receptors, carvedilol decreases the intrahepatic resistance and enhances the reduction of portal pressure and activated HABR (Fig. 6 box 2) [11, 40].

Publications from 1987 to date describe the reduction of PVP by administration of carvedilol in patients with cirrhosis [10, 41–48]. Similarly, a decrease of hepatic venous pressure gradient was reported at 1 h after the administration of carvedilol (see Additional file 1: Table S3) [11, 42–44].

This effect was also reported in an experimental study with cirrhotic rats. This study documented that the administration of 5 mg · kg^−1^ carvedilol by gavage resulted in a significant decrease of PVP from 18,5 ± 0,3 mmHg to 15,2 ± 0,8 mmHg (see Additional file 1: Table S3) [30].

In our study, the administration of carvedilol decreased the PVP in normal rats, but not in rats subjected to the right median hepatic vein ligation + 70% partial hepatectomy.

### Pharmacological modulation of portal hyperperfusion

The pharmacological modulation of portal hyperperfusion was investigated by the administration of terlipressin and octreotide. The pharmacological treatment of portal hyperperfusion in cirrhotic patients is well established as described in many clinical reviews [49–51].

### Terlipressin

The vasopressin analogue terlipressin is a frequently used vasoactive drug to reduce portal hyperperfusion. Terlipressin is a vasoconstrictor and decreases mesenteric and hepatic blood flow. The effect of terlipressin results from the activation of the vasopressin-1 receptors on the smooth muscles of the arterial vasculature in the splanchnic region. This effect should activate HABR as also shown in (Fig. 6 box 2) [13].

Terlipression also leads to a reduction of portal hypertension in cirrhotic patients [52, 53]. Furthermore, terlipressin reduced hepatic venous pressure gradient within 1 to 25 min after injection in cirrhotic patients [54].

When used in an acute experiment with cirrhotic rats, terlipressin reduced portal hypertension during the observation period of 30 min [55]. The administration of the same dose of 0.05 mg · kg^−1^ terlipressin also decreased the PVP after portal vein ligation or partial hepatectomy (80-90% liver resection) during a short observation period of 5 min to 2 h [56–58]. The modulation of HABR by terlipressin was associated with an increase of liver cell proliferation 48 h post-OP after 80% partial hepatectomy in mice [58].

However, in our study, the administration of terlipressin had no effect on modulation of hepatic hemodynamics 24 h after right median hepatic vein ligation + 70% partial hepatectomy.

### Octreotide

The long-acting somatostatin analogue octreotide is also a frequently used vasoactive drug to reduce the portal hyperperfusion [14]. Octreotide inhibits gastrointestinal vasodilators such as glucagon and insulin and enhances the effect of gastrointestinal vasoconstrictors such as protein kinase C. Both together lead to a decrease of splanchnic perfusion and subsequently of portal venous flow to the liver. This effect may also activate the HABR (Fig. 6 box 2) [59].

Hwang already reported in 1992, that octreotide was administered to patients with cirrhosis and variceal hemorrhage [60].

In animal models of portal hypertension, the administration of octreotide decreased portal pressure in acute experiments with a short observation time between 20 min and 1 h [56, 61–64]. Furthermore, Fort et al. reported that the administration of octreotide in a dose of 10 μg · kg^−1^ by s.c. injection prevented the development of hepatocellular necrosis induced by carbon tetrachloride in rats [65]. This renders this substance an interesting drug candidate to decrease the large confluent hepatocellular necrosis after right median hepatic vein ligation + 70% partial hepatectomy [4].

However, in our study, the administration of octreotide had no effect, neither on modulation of hepatic hemodynamics 24 h nor on the severity of confluent necrosis.

### Pharmacological modulation of arterial hypoperfusion

#### Sildenafil

The cGMP specific phosphodiesterase-5 inhibitor sildenafil was used for pharmacological modulation of arterial hypoperfusion. Sildenafil inhibits the degradation of the vasodilative compound cyclic guanosine monophosphate (cGMP). Downstream in the NO pathway, cGMP leads to vasodilatation (Fig. 6 box 1) [15].

The drug is clinically well known for the treatment of erectile dysfunction and the treatment of pulmonary arterial hypertension [66–68].

Halverscheid et al. reported that the intravenous injection of 10 μg · kg^−1^ sildenafil increased hepatic arterial flow in untreated rats within a few min and that the effect lasted for at least 60 min [69]. Yardimci et al. showed that the intraperitoneal administration of 10 μg · kg^−1^ sildenafil improved hepatocyte proliferation 1d post-OP and liver weight recovery 5d after 70% partial hepatectomy [70].

However, in our study, sildenafil did not affect hepatic hemodynamics 24 h after right median hepatic vein ligation + 70% partial hepatectomy.

As outlined above, splenectomy and all drugs were selected on solid grounds, but did not fulfill our expectations towards the modulation of hepatic hemodynamic and the reduction of hepatocellular damage.

The seemingly contradictory observations in respect to hepatic hemodynamics could possibly be attributed due to different experimental conditions. On the one hand the experimental design of other studies showed substantial differences in the over-all observation time. On the other hand, the design differed in respect to the extent of liver resection respectively the loss of functional liver mass. The effect of all these drugs on hepatic hemodynamics was frequently measured in acute experiments of less than 8 h observation time and rarely over an extended observation period as in our study. In our experiments, splenectomy reduced PVF and the application of ISMN reduced PVP immediately after extended liver resection. However, neither splenectomy nor any of the investigated drugs had a long-lasting effect on hepatic hemodynamics and on hepatic damage despite the modulatory effect immediately after the surgical intervention. Furthermore, our own study as well as our literature review suggested that effects of the therapeutic interventions occurred more frequently in case of rather large liver resection exceeding at least 80% of the liver mass. Our surgical procedure caused a surgical loss of approximately 70% of the liver mass plus a functional loss of maximally 10% due to confluent necrosis in the outflow obstructed right median liver lobe.

Nevertheless, the need for a therapeutic intervention to decrease outflow obstruction induced hepatic damage after extended liver resection remains. Based on our experiences, selecting a promising drug candidate remains a challenge in view of the multitude of drugs within the 20 classes of drugs we identified in our literature work up. Identification of the most relevant and potentially druggable pathway is needed. This selection could be supported by a bioinformatic analysis of gene expression.

## Limitations

The study aimed for investigating the effect of pharmacological modulation of hepatic hemodynamics on the extent of hepatic damage developing after focal hepatic outflow obstruction. We limited our investigations to hepatic hemodynamics and hepatic damage as primary read-out parameter. Further assessment of tissue or systemic oxygenation could contribute to a better understanding of tissue hypoxia as a major factor determining the overall damage to the liver.

Furthermore, we limited our focus on two most important observation time points, immediately after the surgical procedure to evaluate the effect of the drugs on hepatic hemodynamics and 24 h later to assess the consecutive hepatic damage.

Adding a prolonged observation phase immediately after the procedure might help to better characterize the underlying hemodynamic adjustment. Adding later observation time points (3d, 5d, 7d) could help to better investigate the effect of the drugs on the kinetics of the further recovery and regeneration process.

## Conclusion

Based on previous studies, we hypothesized that the recovery of parenchymal necrotic areas in the OZ after the right median hepatic vein ligation is dependent on the balance between PVF and HAF. Knowledge-based selection of the five drugs acting on different molecular pathways did not result in the reduction of hepatic damage despite the modulation of hepatic hemodynamics. Selection of other drug candidates from the 20 drug classes could be supported by a bioinformatic analysis of gene expression to identify the involved relevant and potentially druggable pathways.

## Additional file

Additional file 1: Table S1. Effect of splenectomy on hepatic hemodynamics immediately after surgical intervention. Presents results of portal vein pressure, portal flow and hepatic arterial flow measurements of comparable publications. **Table S2.** Effect of isosorbide-5-mononitrate. Presents results of portal vein pressure and other measurements after the administration of isosorbide-5-mononitrate of comparable publications. **Table S3.** Effect of carvedilol. Presents results of portal vein pressure measurements after the administration of carvedilol of comparable publications. (DOCX 25 kb)

## Abbreviations

ALAT: Alanine transaminase
ASAT: Aspartate transaminase
BrdU: 5-bromo-2-deoxyuridine
BZ: Border zone
cGMP: Cyclic guanosine monophosphate
FHOO: Focal hepatic venous outflow obstruction
HAF: Hepatic arterial flow
HE: Haematoxylin-eosin
HVPG: Hepatic venous pressure gradient
ISMN: Isosorbide-5-mononitrate
L-NAME: L-N^G^-Nitroarginine methyl ester
LW/BW: Liver weight-to-body weight ratio
MAP: Mean arterial pressure
NO: Nitric oxide
NZ: Normal zone
OZ: Obstruction zone
pHx: Partial hepatectomy
PVF: Portal flow
PVP: Portal vein pressure
RMHV: Right median hepatic vein
SD: Standard deviation
