# Supplementary material for: Modulation of hepatic perfusion did not improve recovery from hepatic outflow obstruction
Source: BMC Pharmacol Toxicol. 2017 Jun 26;18:50. doi: 10.1186/s40360-017-0155-4 (PMC5485608; doi:10.1186/s40360-017-0155-4)
Supplement: Additional file 1: Table S1. — Effect of splenectomy on hepatic hemodynamics immediately after surgical intervention. Presents results of portal vein pressure, portal flow and hepatic arterial flow measurements of comparable publications. Table S2. Effect of isosorbide-5-mononitrate. Presents results of portal vein pressure and other measurements after the administration of isosorbide-5-mononitrate of comparable publications. Table S3. Effect of carvedilol. Presents results of portal vein pressure measurements after the administration of carvedilol of comparable publications. (DOCX 25 kb) [file 40360_2017_155_MOESM1_ESM.docx]

**Table S1 Effect of splenectomy on hepatic hemodynamics immediately after surgical intervention**

| **Parameter** | **Species** | **Intervention** | **Remnant liver mass** | | | | | | | | | | | | | | | **Reference** |
| --- | --- | --- | --- | --- | --- | --- | --- | --- | --- | --- | --- | --- | --- | --- | --- | --- | --- | --- |
|  |  |  | **70%** | | | | **30%** | | | | **20-15%** | | | | **10%** | | |  |
|  |  |  | **Control** | **Spl** | | | **Control** | | **Spl** | | **Control** | | **Spl** | | **Control** | | **Spl** |  |
| PVP or HVPG [mmHg] | rats  n=5 | LDLT | 11,2±2,4 | 8,0±1* | | |  | | | |  | |  | |  | | | 27 |
|  | rats  n=4 | OLT |  |  | | |  | |  | | 16,0±1,0 | | 12,0±0,5* | |  | |  | 28 |
|  | dogs  n=10 | pHx |  | |  | |  | |  | | 16,4±1,2 | | 17,1±1,0 | |  | |  | 21 |
|  | swine  n=3-4 | pHx |  | |  | | 4,1^# G^  (2,2–5,9) | | 2,5^#^  (1,6–4,2) | |  | |  | | 6,3^# G^  (5,9–9,2) | | 7,5^#^  (6,4–9,9) | 22 |
|  | rats  (n=4) | RMHVL+ 70% pHx |  | | | | 15,4±4,4 | | 14,3±4,4 | |  | |  | |  | |  | current study |
| PVF  [ml/min · g] | rats  n=10 | pHx |  | | |  | |  | |  | |  | |  | | 5,4±0,4 | 3,5±0,4* | 20 |
|  | rats  n=5 | pHx | 1,3±0,5 | | 1,0±0,2 | | 3,5±1,0 | | 2,5±0,5 | | 4,0±1,0 | | 3,2±0,5 | | 5,5±1,0 | | 4,5±1,0 | 23 |
|  | dogs  n=10 | pHx |  | |  | |  | |  | | 3,6±0,3 | | 3,7±0,2 | |  | |  | 21 |
|  | swine n=3-4 | pHx |  | |  | | 2,3^# G^  (2,0–2,8) | | 2,3^#^  (1,7–2,7) | |  | |  | | 3,4^# G^  (3,0–4,5) | | 3,4^#^  (3,2–4,7) | 22 |
|  | rats  (n=4) | RMHVL+ 70% pHx |  | |  | | 1,2±0,4 | | 0,9±0,2* | |  | | | |  | |  | current study |
| HAF [ml/min · g] | rats  n=5 | pHx | 0,2±0,0 | | 0,2±0,1 | | 0,2±0,1 | | 0,3±0,1 | | 0,2±0,1 | | 0,4±0,1* | | 0,2±0,1 | | 0,6±0,1* | 23 |
|  | swine n=3-4 | pHx |  | |  | | 0,3^# G^  (0,2–0,3) | | 0,2^#^  (0,2–0,3) | |  | |  | | 0,1^# G^  (0,1–0,2) | | 0,1^#^  (0,1–0,2) | 22 |
|  | rats  (n=4) | RMHVL+ 70% pHx |  | | | | 0,2±0,1 | | 0,1±0,1 | |  | | | |  | |  | current study |

Control: different animals in control group and treatment group; Spl: splenectomy; OLT-partial orthotopic liver transplantation;

LDLT-living donor liver transplantation; RMHVL-RMHV ligation, pHx – partial hepatectomy

HVPG - hepatic venous pressure gradient

^G^ same animals in control group and treatment group

^#^ median

* P < 0.05 compared to control

**Table S2** **Effect of isosorbide-5-mononitrate**

| **Disease** | **Types** | **Dose of**  **isosorbide-5-mononitrate** | **Observation time** | **Effect on portal pressure or HVPG** | | **Other effects** | **Reference** |  |
| --- | --- | --- | --- | --- | --- | --- | --- | --- |
|  |  |  |  | **Before treatment**  **[mmHg]** | **After treatment**  **[mmHg]** |  |  |  |
| cirrhosis | patients  n=9-10 | 20 mg or 40 mg/patient oral  (0,3mg/kg or 0,6mg/kg^W^) | 2h | 0,3mg/kg: -10%  0,6mg/kg: -18% | |  | 12 |  |
| cirrhosis | patients  n=12 | 40 mg/patient oral  (0,6mg/kg^W^) | 40min | 20,0±4,0 | 18,0±4,7* |  | 36 |  |
| cirrhosis | patients  n=21 | 20 mg/patient oral  (0,3mg/kg^W^) | 1h | 18,4±0,9 | 16,5±0,9* |  | 37 |  |
| cirrhosis | patients  n=12 | 10 mg or 40 mg/patient oral  (0,1mg/kg or 0,6mg/kg^W^) | 1h | 15,5±2,0 | 12,5±1,5* |  | 39 |  |
| cirrhosis | patients  n=11 | 40 mg/patient oral  (0,6mg/kg^W^) | 3 months | 18,6±3,4 | 17,2±3,1* |  | 38 |  |
| cirrhosis model (carbon tetrachloride) | rats  n=6 | 1,8; 3,6 or 7,2mg/kg  oral | 15 days |  |  | 7,2mg/kg: reduce fibrose | 31 |  |
| RMHV ligation + 70% pHx | rats  (n=4) | 7,2mg/kg·12h^−1^  by gastric gavage | 0h | 15,4±4,4 | 9,6±2,3 |  | current study |  |

HVPG - hepatic venous pressure gradient, RMHVL-RMHV ligation, pHx – partial hepatectomy

^W^ Dosis per mg/kg based von mean patient weight from the publication or estimate patient weight of 70kg

* P < 0.05 compared to before treatment group

**Table S3 Effect of carvedilol**

| **Disease** | **Types** | **Dose of carvedilol** | **Observation time** | **Effect on portal pressure or HVPG** | | **Reference** |
| --- | --- | --- | --- | --- | --- | --- |
|  |  |  |  | **Before treatment**  **[mmHg]** | **After treatment**  **[mmHg]** |  |
| cirrhosis | patients  n=10 | 12.5mg/patient oral  (0,2mg/kg ^W^) | 1h | 16,4±0,7 | 12, 6±1,3* | 11 |
| cirrhosis | patients n=16 | 25 mg/patient oral  (0,4mg/kg ^W^) | 1h | 16,7±0,9 | 13,5±1,0* | 42 |
| cirrhosis | patients  n=14 | 25 mg/patient oral  (0,4mg/kg ^W^) | 1h | 19,5±1,3 | 15,4±1,0* | 43 |
| cirrhosis | patients  n=10 | 25 mg/patient oral  (0,4mg/kg ^W^) | 1h | 22,2±4,4 | 15,2±3,7* | 44 |
| cirrhosis | patients  n=21 | 14mg/patient oral  (0,2mg/kg^W^) | 1,5h | 17,6±4,2 | 12,7±2,8* | 45 |
| cirrhosis | patients n=11 | 25mg/patient oral  (0,4mg/kg^W^) | 1,5h | 18,9±1,8 | 15,6±1,9* | 46 |
| cirrhosis | patients  n=26 | 31mg/patient oral  (0,4mg/kg^W^) | 11,1±4,1weeks | 19,0±1,1 | 15,2±0,8* | 47 |
| cirrhosis | patients  n=70 | 3-12,5mg/patient ·12h^−1^ oral  (0,04-0,2mg/kg^W^ ·12h^−1^) | 12 month | 14.5±4.3 | 13.4±5.8 | 48 |
| cirrhosis model (common bile duct ligation) | rats  n=9 | 5mg/kg·12h^−1^  by gastric gavage | 1 week | 18,5±0,3^G^ | 15,2±0,8* | 30 |
| RMHV ligation + 70% pHx | rats  (n=4) | 5mg/kg·12h^−1^  by gastric gavage | 0h | 15,4±4,4^G^ | 12,0±0,6 | current study |

HVPG - hepatic venous pressure gradient, RMHVL-RMHV ligation, pHx – partial hepatectomy

^W^ Dosis per mg/kg based von mean patient weight from the publication or estimate patient weight of 70kg

^G^ different animals in control group and treatment group

* P < 0.05 compared to before treatment group
